# Exploring the effect of perceived overqualification on knowledge hiding: The role of psychological capital and person-organization fit

**DOI:** 10.3389/fpsyg.2022.955661

**Published:** 2022-08-18

**Authors:** Jing Zhu, Fangyu Lin, Ying Zhang, Shanshan Wang, Wenxing Tao, Zhenyong Zhang

**Affiliations:** ^1^Huazhong University of Science and Technology, Union Shenzhen Hospital, Shenzhen, China; ^2^Swinburne University of Technology, Hawthorn, VIC, Australia; ^3^Yunnan University of Finance and Economics, Kunming, China; ^4^Charles Sturt University, Bathurst, NSW, Australia; ^5^Dhurakij Pundit University, Bangkok, Thailand; ^6^The First People's Hospital of Yunnan Province, The Affiliated Hospital of Kunming University of Science and Technology, Kunming, China

**Keywords:** perceived overqualification, knowledge hiding, psychological capital, person-organization fit, organization climate

## Abstract

Individuals' knowledge hiding behavior may lead to massive economic losses to organizations, and exploring the antecedents of it has crucial relevance for mitigating its negative influences. This research aims to investigate the impact of perceived overqualification on knowledge hiding by testing the mediating effect of psychological capital and the moderating effect of person-organization fit. Empirical analyses were conducted on 249 employee dataset using versions SPSS 26 and AMOS 26. Results illustrate an inverse correlation between perceived overqualification and knowledge hiding behavior which is partly mediated by psychological capital and moderated by person-organization fit, implying that good organizational atmosphere that builds up individual psychological capital with better person-organization fit will allow employees to work positively to reduce knowledge hiding behavior when perceived overqualified. This study complements a small quantity of discussions on the positive impact of perceived overqualification on knowledge management and fills omissions in previous studies on the negative effect of perceived overqualification on knowledge hiding behavior in changing surroundings.

## Introduction

Previous studies illustrate that knowledge sharing has been an important asset for organizations to innovate (e.g., Toma and Butera, 2009; Men et al., 2020; Ma and Zhang, 2021; Lee et al., 2022). However, in spite of organizations' effort to forming an atmosphere that will actively disseminate information, the phenomenon of knowledge hiding (KH) practices in organizations is not rare (Connelly et al., 2019; Men et al., 2020; Lee et al., 2022). Due to the universality and prevalence of knowledge hiding (Zhang and Min, 2021), exploring its antecedents is crucial to preventing management problems. Since the establishment of the knowledge hiding structure in 2012, a great deal of researches has investigated the causes and consequences of this behavior to reduce or prevent it (Connelly et al., 2019). Existing research has focused on increasing knowledge hiding behavior in times of high distrust and competition (Hernaus et al., 2018). Knowledge hiding has enormous effects on organizations, relationships, and individuals, and is associated with poor employees' innovation and performance (Černe et al., 2014; Rhee and Choi, 2017; Babič et al., 2018). Since employees are the critical carriers of organizational knowledge, recruiting excellent talents with higher knowledge level is a way for many enterprises to acquire knowledge (Capsada-Munsech, 2019). That is, the strict requirements for job seekers (Fine and Edward, 2017) and the training for employees can contribute to the knowledge level (Wang and Noe, 2010). Nevertheless, everything has double effects. These steps may raise the literacy of the enterprise, but it has the tendency of increasing perceived overqualification (POQ).

Findings of existing research on the perceived overqualification are mixed. A large number of the studies focused on the harmful consequences of negative psychological effect of perceived overqualification (e.g., McKee-Ryan and Harvey, 2011; Zhang et al., 2016). This is because perceived overqualification is widely considered to be contributing to inactive psychology. For example, perceived excess qualification may increase employees' sense of job alienation and weaken their organizational self-esteem (Zhao et al., 2019); it may also well reduce employees' intrinsic motivation (Chen et al., 2017), thereby reducing role behavior, thus may increase the likelihood of knowledge hiding. Nonetheless, the potential constructive aspects of perceived overqualification have been largely overlooked and only a few empirical studies examined the positive effect of perceived overqualification on employees, discovering that perceived overqualification can shape an individual's positive psychological cognition, such as task mastery, role self-efficacy and work engagement (e.g., Deng et al., 2018; Erdogan et al., 2020; Ma et al., 2020). Drawn from the theory of psychological capital, perceived overqualification is far less negative for two main reasons: First, employees with overqualification tend to have a higher level of knowledge (Erdogan et al., 2020), and they are more likely to spread and share information. Indeed, sharing information may well combat possible pressure and frustration generated from perceived overqualification and make employees obtain higher social recognition, thereby accumulate social capital. Second, employees with excess qualifications may also have a greater sense of psychological security (Den Hartog and Belschak, 2012). That is, qualifications and experience can enhance the sense of self-efficacy, conducive to divergent thinking in the process of solving problems, and breaking the existing cognitive and thinking constraints, which are likely to reduce knowledge concealment effectively. It is apparent that conflicting perspectives are found in the above-mentioned studies, and both views mentioned the role of individual psychological factors in the relationship between perceived excess qualification and knowledge concealment. The former emphasizes the mediation of positive psychological capital, while the latter focuses more on the role of negative psychology itself, and both need to be considered. While acknowledging the negativity of perceived overqualification, this study responds to the call for further exploring the potential positive outcomes of the perceived overqualification, such as lower levels of knowledge hiding.

Since the relationships between these interactions may be complex and depend on the various situations, we test its effect on knowledge hiding with critical consideration given to the role of psychological capital and person-organization fit, aiming to validate and excavate the potential affirmative effect of perceived overqualification and its impending encouraging outcomes, which were largely ignored in a large number of previous studies. Moreover, in organizational knowledge management process, person-organization fit (P-O fit) continues to act as a transmitter by linking itself to organizational atmosphere and values and helping organizations overcome difficulties or challenges. Studies (e.g., Erdogan et al., 2011; Zhao et al., 2021) have shown that the effectiveness of person-organization fit depends on the similarity or consistency between organizational and individual values, interests, beliefs, and needs. With higher degree of personal organizational fit, employees with excess qualifications are more likely to actively participate in behaviors beneficial to colleagues (such as advice behavior, organizational citizenship behavior to others), thereby obtain higher advice network centrality to improve their position in the social network (Erdogan et al., 2020). Drawn from the theory of person-environment fit (Shalley et al., 2000), the individual and environment fit helps to stimulate positive personal attitudes and behaviors. That is, the supportive culture and atmosphere of the organization not only predict the innovative behavior of individuals but also form an organizational ambiance of trust and cooperation (Collins and Smith, 2006). Therefore, the person-organization fit may have a certain effect on regulating the leading causes of knowledge hiding behavior. It encourages, motivates, and drives individuals to actively engage in the organization. Despite these benefits, few studies have understood how the match between individuals and organizations affects individual knowledge hiding behavior. As far as we know, the moderating effect of person-organization fit on knowledge hiding behavior has not been empirically explored so far. Therefore, this study is designed to answer three sets of questions: (i) Does the perceived overqualification affect knowledge hiding, and how? (ii) Does psychological capital actively mediate the association between the perceived excess qualification and knowledge hiding? (iii) Does person-organization fit moderate the association between the perceived overqualification and knowledge hiding? Overall, this exploration aims to contribute to the literature on psychological capital and knowledge management. Firstly, through examining the role of the perceived overqualification in predicting often overlooked potentially positive aspects of knowledge hiding behavior, our study would enrich existing research and deepen our understanding of the subject of how individuals' psychological capital, person-organizational fit, and overqualification affect the process of knowledge hiding. Secondly, based on previous studies on the emotional reasons for knowledge concealment in organizations, the mediation effect of psychological capital on knowledge hiding is tested. Third, this study may well extend our understanding of the potential mechanisms underlying the interaction between person-organization fit and knowledge hiding behavior by demonstrating the regulatory role of person-organization fit. Finally, this study provides practical advice for managers to prevent unnecessary individual knowledge hiding.

The rest of the paper is arranged as follows. Section Literature and hypotheses presents the literature review and hypothetical development. Section Materials and methods describes the research methodology. Section Results details data analysis and results. Section Discussion addresses discussion and implications, and section Conclusion details the contributions of this paper and the limitations of our study and future research directions.

## Literature and hypotheses

### Perceived overqualification and knowledge hiding

In the changeable information age, many corporations have focused more prominently on the use of knowledge (Liu et al., 2018; Ganguly et al., 2019). Knowledge sharing is the key to knowledge management (Wang and Noe, 2010; Lei et al., 2019), the main problem of current knowledge management is how to facilitate information transmission among individuals and reduce knowledge hiding (King and Marks, 2008; Yao et al., 2020). This is crucial because knowledge cannot be fully utilized, which may affect the performance of the organization (Carmeli et al., 2011; Zhang et al., 2021b). Connelly et al. (2012) define knowledge hiding as “individuals intentionally conceal or hide the knowledge requested by others”. That is, knowledge hiding can be deemed as a strategy for employees to form a competitive advantage and maximize individual benefits in an organization. Importantly, in highly competitive environments, employees' knowledge hiding behavior will not only have a potential negative impact on employees themselves but also be a major threat to the development of contemporary enterprises, which may lead to massive economic losses to the organization (Černe et al., 2014; Connelly et al., 2019; Zhang et al., 2022). Despite the importance of knowledge hiding, there is little literature on the subject. Based on the significant benefits of knowledge, enterprises will pay more attention to the acquisition and effective use of information. Researchers have mainly categorized knowledge hiding in three elements. Connelly and Zweig (2015) indicates that knowledge hiding is individuals intentionally hiding or concealing knowledge requested by others and proves that knowledge hiding consists of three independent but related elements: i) Rationalized hiding, i.e., the concealer justifies, by implying that the requester is unable to provide the requested knowledge; ii) Playing dumb, i.e., the concealer pretends not to know the relevant information or knowledge; iii) Evasive hiding, i.e., the concealer provides incorrect information or knowledge.

Overqualification stems from the concept of underemployment and is a sub-dimension of it (Feldman et al., 2002). Overqualification is divided into objective and subjective. Objective overqualification means that the knowledge, skills, and abilities (KSAs) that an individual possesses more than the basic requirements and work experience of the job position (Maynard et al., 2006; Erdogan et al., 2011). It is determined by contrasting whether the individuals' qualifications are in line with the work label (Martinez et al., 2014; Zhang et al., 2016; Sesen and Ertan, 2019). This is an objective fact, assessed fairly and impartially by an observer who is not subject to external constraints (Maltarich et al., 2011; Sesen and Ertan, 2019) and only used to understand the psychological experiences of employees who have overqualifications (Erdogan et al., 2011; Chu et al., 2021). Subjective overqualification is described as “the degree to which individuals perceive their qualifications to match the position requirements” (Maynard et al., 2006). The perceived overqualification is related to individuals' personal opinions about them (Sesen and Ertan, 2019), within the category of subjective evaluation. Studies show that individuals with subjective overqualification are more susceptible to the mismatch between their surroundings and their eligibility (Fine, 2007; Luksyte et al., 2011; Chu et al., 2021).

This paper examines perceived overqualification and its effect on knowledge management, and therefore emphatically discusses the concept of the perceived overqualification. Previous studies found positive and negative regarding the consequences of the perceived overqualification, and maybe the individuals can treat and respond to their views of the perceived overqualification from two different points. Different individual abilities generate different effects on their work performance (Zhang et al., 2021c). Firstly, they may feel that their capacity is underutilized. This psychological feeling may cause them to have negative emotions, which in turn change their positive attitude and correct behavior (Maynard et al., 2006; Erdogan and Bauer, 2009; Chu et al., 2021). Most studies often adopt these ideas, such as affected individuals may develop negative emotions and psychology (Peiró et al., 2012; Liu et al., 2015; Simon et al., 2019), reducing work pleasure and increase the willingness to leave (Erdogan et al., 2020; Wu and Chi, 2020).

Previous studies have stressed on work behavior under the influence of individuals' perceived overqualification with different conclusions. Most studies have argued that the perceived overqualification can adversely affect individuals' knowledge management behaviors, such as knowledge hiding (Khan et al., 2022; Li C. S. et al., 2022). Zutshi et al. (2021) believe that knowledge hiding is more correlated to negative emotions. Some researchers have also shown that perceived overqualification reduces knowledge-sharing behavior. Comparing knowledge hiding and knowledge sharing, deliberate attempts to hide information that another person needs is called knowledge hiding (Li C. S. et al., 2022). The researchers highlight the main difference between the two behaviors (sharing and hiding) may be their motive (Agarwal et al., 2021; Zhong et al., 2021; Khan et al., 2022). In prior studies, some scholars have proposed that the perceived overqualification of individuals was positively associated with knowledge management behavior of individuals in organizations. Employees perceived overqualification enhances their proactive work behavior (Erdogan et al., 2011; Zhang et al., 2016), and they may be involved in the work formulation actively (Maynard, 2011; Russell et al., 2016; Lin et al., 2017; Sesen and Ertan, 2019). Existing studies have confirmed that employees who perceived higher overqualification prefer to express their personal views in the organization to gain recognition. When individuals have the remaining KSAs in their work (At least in their self-perception), they may take a positive approach, i.e., they are willing to use the remaining KSAs to help colleagues (Li C. S. et al., 2022). Individuals with stronger KSAs often better understand, interpret, and use a variety of information (Earley, 2002; Earley and Peterson, 2004; Zhang Y. et al., 2020). For example, teams and organizations may request knowledge from the individuals who perceived overqualification by encouraging the others or leverage the KSAs of perceived overqualification individuals by having overqualified individuals guide more capable colleagues and impart their knowledge across the team (Thompson et al., 2013; Russell et al., 2016; Sikora et al., 2016). It enables individuals to obtain the satisfaction of self-worth, and the team or organization maximizes the benefits of individuals. Current empirical research on perceived excess of qualification and knowledge concealment is inadequate. We believe that the individuals who feel good about themselves will show their knowledge advantages and abilities to others in a positive way, so, according to the above literature review, we make the following hypothesis:

H1: Perceived overqualification is negatively associated with knowledge hiding.

### The mediation role of psychological capital

Psychological capital (PsyCap) is described as a developmental state of a person's positive psychology, including optimism, hope, resilience, and self-efficacy (Luthans et al., 2007; Avey et al., 2011), which can be established to improve their performance (Luthans, 2002; Luthans and Youssef, 2004; Luthans et al., 2007). The psychological capital appears to capture an individual's psychological abilities, containing four dimensions that all represent a positive assessment of a person's abilities. As an important psychological ability for individuals to undertake more demanding tasks (Parker and Collins, 2010), psychological capital is a primary component to study the relationship between the main variables (POQ and KH) studied in this paper. To some extent, the perceived overqualification is central to the formation of individual psychological capital (Gist and Mitchell, 1992; Den Hartog and Belschak, 2012). For example, when analyzing task requirements, individuals with higher qualifications may consider their tasks relatively easy and can be performed effortlessly, however, owing to possessing more resources (such as KSAs), individuals rather seek a wider range of tasks actively than instructions limited to upper managers, and they will better master additional tasks beyond their job requirements or responsibilities. In other words, individuals with surplus KSAs are more likely to perform challenging tasks and achieve these goals confidently, which leads to a higher assessment of their psychological capital. In a similar vein, Jung and Yoon (2015) believe that whether individuals can perform tasks within or outside their roles excellently depends on their psychological ability to a certain extent. Psychological ability will affect their subjective initiative and reduce the passive attitude of individuals in the organization. Therefore, psychological capital plays an important part in knowledge management, that is, individuals' optimism, hope, resilience, and self-efficacy, which provide psychological safety and psychological support for reducing the hidden behaviors of individuals' knowledge and enhancing other knowledge behaviors such as the sharing and application of individuals' KSAs.

Drawn from psychological resource theory, psychological capital is a high-order structure consistent with active organizational behavior (Luthans and Youssef, 2004), and existing research focuses on its impact on organizational management. Psychological capital is a basic concept of positive organizational behavior (Avey et al., 2009), which emphasizes motivational rather than negative factors. In this context, psychological capital promotes job performance and job creation that are conducive for individuals to control their emotions and cope with stress, and reduce their negative behaviors (Avey et al., 2009). Studies have shown that skill utilization is positively associated with self-esteem in the workplace (Aryee and Luk, 1996). In particular, individuals who perceive overqualification may simultaneously provide dynamic energy in the whole process, with their own excess KSAs, and actively help colleagues to control the adverse impact of the perceived overqualification in this positive way. Accordingly, based on capability perspective of positive organizational behavior theory, they will use redundant KSAs to help the team and other members of the organization, and then make more contributions to the team or organization. In this process, its KSAs are valued by the organization, which promotes positive self-evaluation, and then affects their self-efficacy (Lai, 2011; Luksyte et al., 2011; Vinayak et al., 2021), forming a favorable cycle between perceived overqualification, psychological capital, and individual knowledge management behavior.

While scholars focus on the importance of individual characteristics, behaviors, and abilities in the context of knowledge management, there are few precedents for psychological capital. As for the potential of psychological capital structure, it has not been fully tested in the existing literature (Zhang et al., 2021a). Indeed, there is rather little literature on the effects of the perceived overqualification on psychological capital. Self-determination theory (Ryan and Deci, 2000) sees the need for competence, relevance, and autonomy as fundamental to a person's mental health and innate motivation. Employees with the self-identified remaining KSAs are more likely to perform challenging tasks. Their qualifications and experience give them a sense of self-efficacy that allows them to make full use of their skills. They may have a better command of other tasks beyond their job requirements or responsibilities, and then meet the need for motivation and internal motivation to work, driving his hope, optimism and resilience formation, which will lead to a higher evaluation of his psychological capital. Therefore, given the importance of the perceived overqualification for knowledge management based on the theory of planned behavior, it is reasonable to predict that perceived overqualification may have a positive effect on individual psychological capital. Consequently, we recommend that:

H2: Perceived overqualification is positively associated with the psychological capital.

Prior research has revealed the difference between information dissemination and knowledge hiding. Connelly et al. (2012) distinguished two kinds of knowledge management behaviors: knowledge sharing (KS) and knowledge hiding (KH). The sharing is “the act of spreading knowledge within an organization” and “the conscious act of a person who knows” (Ipe, 2003). The study focuses on double key sides of sharing: the frequency and the quality or usefulness (Soo et al., 2004; Swift and Virick, 2013; Gagné et al., 2019). Kim and Park (2017) argue that only knowledge can be managed efficiently through effective knowledge sharing.

Though information dissemination and knowledge hiding are usually seen as the contrary behavior, their motives are distinctive (most cases are irrelevant). In other words, they are relatively independent actions, and also vary with each individual (Gagné et al., 2019). Knowledge hiding is not entirely negative behavior and does not necessarily harm an individual or organization (Connelly and Zweig, 2015). It has been distinguished from many related but different behaviors, such as cheat (Webster et al., 2008). In the light of Connelly et al. (2012), people's knowledge hiding occurs more due to distrust, and this behavior may be influenced by their cognition of organizational and individual psychology. However, when colleagues recognize the occurrence of knowledge hiding behavior, a cycle of mistrust will occur (Akhtar et al., 2021).

According to the social cognitive theory, self-efficacy in psychological capital is the inner driving power for individual initiative. Whether or not a person is willing to do something outside of their job will affect whether or not they decide to take action and invest the effort and time (Hong et al., 2016). The individuals who perceived overqualification feel that they have the remaining KSAs, so they have enough confidence and motivation to help others in the organization or team members, answer their questions, avoid knowledge hiding, and perform work responsibilities beyond their prescribed roles. Individuals with higher psychological capital are more inclined to control their knowledge hiding behavior. In addition, some studies have found that highly confident and optimistic employees can feel motivated and can control their own fate (Alessandri et al., 2018). They are willing to devote the resources and effort and to meet any challenges (Stajkovic and Luthans, 1998). When employees with high psychological capital experience negative experiences at work, they are likely to adjust and bounce back from higher resilience. Finally, the higher the individual hopes, the higher their chances of finding multiple ways of success, and the lower the likelihood of having knowledge hidden. Therefore, employees with higher psychological capital will not avoid knowledge sharing and abandon their goals, but will choose to achieve their desired results in a positive way. Furthermore, we believe that the psychological capital ability of employees provides positive psychological support for their positive behavior of knowledge management and make the following assumptions:

H3: Psychological capital is negatively associated with knowledge hiding.

As mentioned earlier, individual characteristics and abilities are crucial factors influencing knowledge hiding behavior. The adjustment of psychological capital is related to the psychological state of individuals in their environment (Ward and Kennedy, 2001; Fish, 2005). This concept is subjective and related to an individual's emotional state, cognitive perception, and personal characteristic variables (Zhang, 2013). Perceived overqualification is an essential feature of outstanding individuals, and psychological capital is influenced by a wider range of leading factors. Given the complexity of the knowledge hiding formation mechanism, it is reasonable to assume that psychological capital would be influenced by the perceived overqualification and play an intermediary role between perceived excess qualification and knowledge hiding.

Specifically, the individuals who perceived high overqualification can respond to and adapt to different working environments, thus changing their working patterns to be consistent with their unique qualifications. Zhang and Oczkowski (2016) put forward the differences between work adjustment, general living conditions adjustment, and interaction adjustment. We believe that these three capabilities are vital for individuals with perceptible qualifications in the organization, and to a certain extent, they reflect the resilience of employees. Karatepe and Talebzadeh (2016) believe that the perceived overqualification drives individuals to develop a positive work attitude to promote a trusting relationship in coordination with colleagues and the organizations, and then establish optimistic psychological ability, so that employees become proactive and flexible, eliminating the negative emotions caused by their high sense of seniority, which also helps to motivate employees to work more efficiently (Hakanen et al., 2018). With all the foregoing considerations, we believe that individuals perceived overqualification favors the formation of their psychological capital, which would have a further impact on knowledge hiding. Therefore, we recommend that:

H4: Psychological capital plays a mediating role in the negative correlation between employees' perceived overqualification and knowledge hiding.

### The moderating role of person-organization fit

Based on social cognitive theory, individual motivation and initiative are influenced by environmental information. The feedback of an organization to individuals will affects the judgments of self-efficacy and the production of positive behavior (Fugas et al., 2012; Zhang et al., 2014; Javed et al., 2021). Research on personal-organization fit (P-O fit) mainly manifests in the similarity between individuals and organizations. This similarity is the compatibility between personal and organizational characteristics of individuals, for example, whether the values and goals of the organization are in line with those of the individuals (Kristof-Brown et al., 2005; Akhtar et al., 2020). In other words, personal-organization fit indicates the consistency between the values of the individuals and the organization (O'Reilly et al., 1991).

Personal-organization fit is essential for perceiving overqualified individuals, as individuals may actively seek out aspects of the organization that are consistent with their aims, thus buffering the adverse effects of their objective mismatches with their jobs. This is reflected in the high degree of compatibility between the values and cultures of individuals and organizations (Follmer et al., 2018), organizations prefer their capability and values and provide a comfortable working environment for individuals to support their development (Maynard and Parfyonova, 2013). Perceived overqualified individuals appreciate the values and culture of the organization and accept the support of the organization. At this time, the individual reaches an agreement with the organization, forming favorable interactions (Wu and Chi, 2020). Eisenberger et al. (1997) argue that the individuals' perceptions of their qualifications and values are influenced by the level of organizational support. Taller personal-organization fit can make individuals more optimistic, and a large of research shows that personal-organization fit affects individual behavior. Edwards and Cable (2009) found three supportive mechanisms in the process of investigating why personal-organization fit generate positive effects. First, personal-organization fit can enhance the common understanding of right and wrong among individuals, thus strengthening the sense of trust between each other. Second, personal-organization fit can promote individuals' common understanding of the same event, which in turn improves their communication and interaction skills. Third, having common values increases the attractiveness of organizations and their individuals to each other. The higher the personal-organization fit, the perceived overqualified individuals are eager to leverage their perceived remaining skills by helping colleagues. Because of mutual trust, they are more pleased to share knowledge with others, and also increased attention to the internal affairs of the organization. They are more inclined to use the excess KSAs to serve their colleagues (Wu and Chi, 2020). Kristof (1996) pointed out that personal-organization fit refers to the overall environment in which individuals operate in the workplace and covers its various elements—organizational attributes: organizational culture, organizational climate, and norms; personal features: personality, values, goals, and attitudes (Wojtczuk-Turek and Turek, 2016). The “fit” between one's psychological climate and the current organizational atmosphere can represent a potentially important factor in employee job satisfaction (Joyce and Slocum, 1982). If the perceived personal-organization fit is high, then the employee's psychological and organizational atmosphere perception will remain consistent (Conner, 2014).

Further, the literature suggests that the employee psychological capital is positively associated with a perceived organizational support climate (Luthans et al., 2008). They propose that a supportive environment may help the psychological capital to flourish among employees. A supportive environment may make workers more resilient and hopeful, encouraging them to actively respond to setbacks and try new methods in their work. Wang and Xie (2021) also expounded on the effect of organizational atmosphere on employees' knowledge hiding in their research on organizational motivational climate. Organizational motivation atmosphere includes two dimensions: organizational control atmosphere and organizational performance atmosphere (Nerstad et al., 2013), among them, the organizational control atmosphere attaches importance to the sustainable development of individuals, encourages individuals to learn from themselves and colleagues to learn from each other, and establishes the common destiny among employees (Ntoumanis and Biddle, 1999). In the controlled atmosphere of an organization, the standard of employees' success is concerned with self-ability improvement, which encourages colleagues' mutual learning and grow together (Ntoumanis and Biddle, 1999). In this atmosphere, individuals hiding their knowledge is not conducive to group achievement, and individuals does not have to worry about sharing knowledge in the high personal-organization fit and organizational emotion (Černe et al., 2014). As a result, individuals are willing to make progress together by sharing their experiences or insights. In a similar vein, it is easy to establish trust during an interaction, mistrust is a key factor in knowledge concealment in a collective (Connelly et al., 2012), and trust can significantly reduce knowledge hiding behavior among members (Zhao and Xia, 2019), this is consistent with the mechanistic theory of personal-organization fit. According to social information processing theory, an important characteristic of humans is adaptability, and individuals adjust their behaviors and attitudes according to the information provided by their surrounding environment (Salancik and Pfeffer, 1978). Thus, we believe when there are higher levels of personal-organization fit; individuals with higher levels of overqualification are more inclined to share knowledge with others. We put forward the following hypothesis:

H5: The degree of person-organization fit moderates the relationship between perceived overqualification and knowledge hiding behavior.

**Figure 1:**
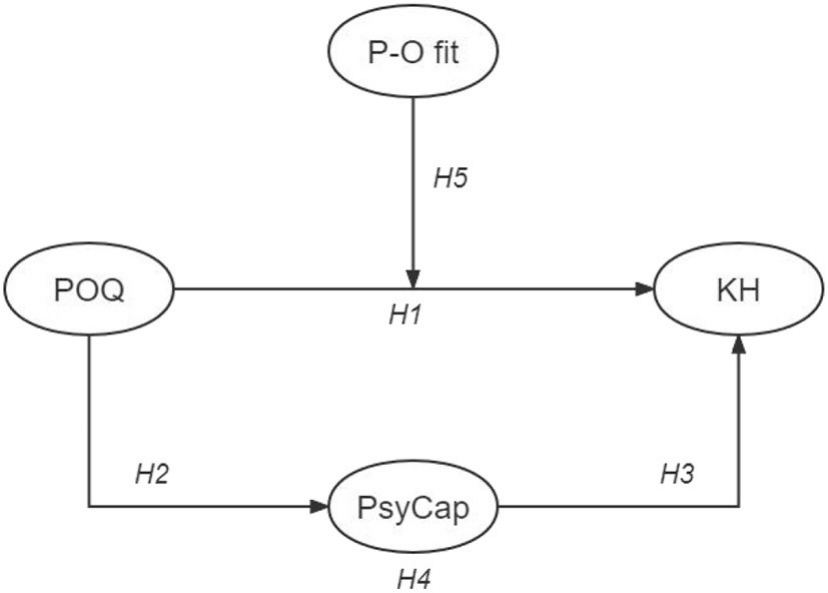
Model of the theoretical framework.

The overall conceptual model is exhibited in Figure 1.

## Materials and methods

### Sample and procedure

Original data was collected for hypothesis testing by survey method based on a network questionnaire. The sample includes organizations in China where knowledge management processes are implemented. The sample population comprises the managers and individuals from state-owned enterprises, foreign-funded enterprises, private enterprises, and government units, all of which are known to be knowledge-intensive industries with knowledge resources and practices according to previous studies.

Before the questionnaire is distributed, as a pilot study, 18 academics from universities in China were invited to evaluate the validity and reliability of the proposed questionnaire. Initially, with the assistance of the Chinese Chamber of Commerce directories, the International Business Cooperation Office based in Kunming, the First People's Hospital of Yunnan Province and Union Shenzhen Hospital, 41 Chinese organizations where knowledge management practices are implemented participated in our survey. To reduce the influence of homologous deviation and clarify the logical relationship between variables, we collected data from the same population at two-time points, with an interval of 3 weeks for each survey in early and late April 2002 respectively. Due to the spread of the COVID-19 pandemic, all data surveys were collected *via* the standardized online platform Microsoft Forms (see questionnaire at http://www.microsoft.com/zh-cn/microsoft-365/online-surveys-polls-quizzes). The survey period was 6 weeks altogether, and a total of 307 responses were obtained, with 9 containing invalid values of more than 25% and 49 cases failed to satisfy the research criteria for the research, resulting in a final data set of 249 usable questionnaires.

### Measures

As detailed below, existing scales from the literature were utilized, and measured on a 5-point Likert-type scale from 1 (strongly disagree) to 5 (strongly agree).

#### Dependent variables

For individuals' knowledge hiding (KH), the KH scale consisting of 12 items advanced by Connelly et al. (2012) was adopted. It contains three dimensions, and each dimension has four items, namely, evasive hiding, playing dumb, and rationalized hiding. It contains three dimensions, and each dimension has four items. A sample item is “Agreed to help him/her but never really intended to.” The Cronbach's alpha reliability is 0.965.

#### Independent variables

For individuals' perceived overqualification (POQ), the POQ scale consisting of 9 items advanced by Maynard et al. (2006) was adopted. A sample item is “Someone with less work experience than myself could do my job just as well.” The Cronbach's alpha reliability is 0.963.

##### Psychological capital (PsyCap)

We measured PsyCap by a 24-item scale advanced by Luthans et al. (2007). It contains three dimensions, and each dimension has four items. Namely, hope, optimism, resilience, and self-efficacy. A sample item is “I feel confident in representing my work area in meetings with management.” The Cronbach's alpha reliability is 0.973.

##### Person-organization fit (P-O fit)

We adopt the scale developed and tested by Cable and DeRue (2002). It is composed of 3 items and has been translated into many languages, and its reliability and validity have been verified by a large number of studies. A sample item is “My organization's values and culture provide a good fit with the things that I value in life.” The Cronbach's alpha reliability is 0.928.

#### Control variables

We control for individuals' age, gender, education level and years of work due to their vital importance on individuals' KH behavior (Ma and Zhang, 2021; Khan et al., 2022). And the industry type, individuals' salary level and individuals' marital status are likely to have an impact on KH (Avey et al., 2010). Responses were coded as: Gender (1 = “Male”, 2 = “Female”); Age (1 = “ <25”, 2 = “26–30”, 3 = “31–40”, 4 = “41–50”); Education level (1 = “Below undergraduate”, 2 = “Undergraduate”, 3 = “Postgraduate”, 4 = “Doctorate and above”); Years of work (1 = “ <1”; 2 = “2-5”; 3 = “6-10”; 4 = “>10”); Industry type (1 = “State-owned”, 2 = “Private”, 3 = “Foreign”, 4 = “Governmental”, 5 = “Others”); Individuals' salary level (1 = “ <3,000”, 2 = “3,001–6,000”, 3 = “6,001–9,000”, 4 = “>9,000”); Individuals' marital status (1 = “Married”, 2 = “Unmarried”, 3 = “Divorce”). The more detailed demographic information is given in Table 1.

**Table 1:** Characteristics of samples.

**Background variables**		**Frequency**	**Percent**
Gender	Male	169	67.9
	Female	80	32.1
Age	<25	50	20.1
	26–30	82	32.9
	31–40	74	29.7
	Above 40	43	17.3
Education	Below undergraduate	77	30.9
	Undergraduate	88	35.3
	Postgraduate	56	22.5
	Doctorate and above	28	11.2
Work years	<2	70	28.1
	2–5	64	25.7
	6–10	53	21.3
	>10	62	24.9
Industry type	Education	54	18.8
	Information	82	28.5
	Manufacturing	89	30.9
	Finance	35	12.2
	others	28	9.7
Salary level	<3,000	27	10.8
	3,001–6,000	90	36.1
	6,001–9,000	86	34.5
	>9,000	46	18.5
Marital status	Married	129	51.8
	Unmarried	104	41.8
	Divorce	16	6.4

### Common method variance

Common method variance (CMV) refers to the variance that is caused by measurement error than to the variable of the study (Bagozzi and Yi, 1991; Syed et al., 2021). It is an organized error variance common among the variables tapped with and emerges as a result of the same source or method used (Richardson et al., 2009). Common method variance generally exists in management studies, although self-assessment does not necessarily exist or create homogeneity, and method variance can deflate or inflate the true relationship between independent and dependent variables (Javed et al., 2020; Akhtar et al., 2022).

Since the data for each item all come from the same respondent, and the results are self-reported, it may lead to a problem of common method variance. Therefore in order to overcome CMV, we used different remedies (Aslam et al., 2021). First, to ensure the realness of the data, we kept the items clear in designing the questionnaire and reiterated that the questionnaire was completely anonymous for distribution. Secondly, our data are obtained from the same population by time and stage. Thirdly, in order to identify the distinctiveness of hypothesized model, we performed confirmatory factor analysis (CFA), where results reveal that (see Table 3) the model fitness of hypothesized model (χ^2^ = 654.572, TLI = 0.952, CFI = 0.957, RMSEA = 0.060) is better than other models. It depicts that common method bias is not a concern in the present study. Finally, after the questionnaire was collected, we used Harman's single factor test (Podsakoff et al., 2003) to evaluate the negative influence of the common method bias. The findings indicated that the first factor without rotation explained 46.82% of the variance, which was <50% of the recommended value. Therefore, the common method bias is unlikely to cause considerable impact (Li C. S. et al., 2022; Li M. et al., 2022).

## Results

### Reliability, convergent, and discriminant validity

#### Reliability analysis

Two sorts of data analysis software namely SPSS and AMOS were used to conduct a preliminary inspection of scales. Cronbach's alpha is used for examining reliability, and Cronbach's alpha of each variable was acceptable and exceeded 0.7 (see Table 2).

**Table 2:** Measurement model reliability and validity analysis results.

**Construct**	**Item**	**Unstandardized estimate**	**S.E**.	***T*-value**	***P***	**Standardized estimate**	**CR**	**AVE**	**Cronbach's α**
POQ	POQ1	1.000				0.884	0.963	0.744	0.963
	POQ2	0.930	0.045	20.664	***	0.886			
	POQ3	0.940	0.048	19.644	***	0.866			
	POQ4	0.931	0.046	20.439	***	0.882			
	POQ5	0.895	0.044	20.354	***	0.880			
	POQ6	0.924	0.047	19.862	***	0.871			
	POQ7	0.907	0.046	19.785	***	0.869			
	POQ8	0.893	0.049	18.376	***	0.839			
	POQ9	0.812	0.050	16.082	***	0.781			
KH	KH1	1.000				0.811	0.965	0.699	0.965
	KH2	0.928	0.067	13.837	***	0.761			
	KH3	1.024	0.060	16.943	***	0.874			
	KH4	0.978	0.061	16.119	***	0.846			
	KH5	0.994	0.058	17.168	***	0.882			
	KH6	0.860	0.060	14.393	***	0.783			
	KH7	1.041	0.064	16.144	***	0.847			
	KH8	0.983	0.058	16.883	***	0.872			
	KH9	0.972	0.060	16.282	***	0.852			
	KH10	0.966	0.060	15.969	***	0.841			
	KH11	0.926	0.060	15.349	***	0.819			
	KH12	1.003	0.063	15.812	***	0.836			
PsyCap	PsyCap1	1.000				0.745	0.973	0.599	0.973
	PsyCap2	1.037	0.084	12.409	***	0.753			
	PsyCap3	0.983	0.083	11.910	***	0.726			
	PsyCap4	0.923	0.079	11.625	***	0.711			
	PsyCap5	1.088	0.085	12.755	***	0.772			
	PsyCap6	1.051	0.083	12.684	***	0.768			
	PsyCap7	0.964	0.080	12.107	***	0.737			
	PsyCap8	0.974	0.079	12.263	***	0.746			
	PsyCap9	1.020	0.080	12.759	***	0.772			
	PsyCap10	1.061	0.084	12.597	***	0.764			
	PsyCap11	0.989	0.078	12.617	***	0.765			
	PsyCap12	0.992	0.076	13.083	***	0.790			
	PsyCap13	1.036	0.078	13.326	***	0.802			
	PsyCap14	1.178	0.086	13.662	***	0.820			
	PsyCap15	1.109	0.081	13.626	***	0.818			
	PsyCap16	1.132	0.085	13.327	***	0.802			
	PsyCap17	0.916	0.077	11.892	***	0.725			
	PsyCap18	1.027	0.080	12.797	***	0.774			
	PsyCap19	1.171	0.085	13.719	***	0.823			
	PsyCap20	0.990	0.078	12.722	***	0.770			
	PsyCap21	1.105	0.084	13.210	***	0.796			
	PsyCap22	1.036	0.079	13.176	***	0.795			
	PsyCap23	1.186	0.085	13.887	***	0.832			
	PsyCap24	0.997	0.081	12.325	***	0.749			
P-O fit	P-O fit1	1.000				0.881	0.928	0.812	0.928
	P-O fit2	1.032	0.050	20.546	***	0.914			
	P-O fit3	0.983	0.048	20.343	***	0.908			

POQ, perceived overqualification; KH, knowledge hiding; PsyCap, psychological capital; P-O fit, person-organization fit. ^***^p < 0.001.

#### Convergent and discriminant validity

Amos was applied to examine the model's convergent and discriminant validity, respectively. For convergence validity, the standardized factor loading, composite reliability (CR), and average variance extracted (AVE) were evaluated (Hair et al., 2009). After testing, the standardized factor loadings in each item were over 0.7, the CR of each dimension was over 0.8 and the AVE was >0.5, which all indicate that convergence validity was acceptable (see Table 2).

In order to evaluate the discriminant validity between POQ, PsyCap, P-O fit and KH, confirmatory factor analysis was applied by Amos. Comparing the resulting data, fit index of four-factor measurement model (χ^2^ = 654.572, *df* = 344, χ^2^/*df* = 1.903, IFI = 0.957, TLI = 0.952, CFI = 0.957, RMSEA = 0.060, RMR = 0.050) was the optimal models (see Table 3), which confirm discriminant validity was acceptable.

**Table 3:** CFA model fit indices.

**Measurement model**	**χ^2^**	**df**	**χ^2^/*df***	**IFI**	**TLI**	**CFI**	**RMSEA**	**RMR**
1 Four-factor model	654.572	344	1.903	0.957	0.952	0.957	0.060	0.050
2 Three-factor model	902.784	149	6.059	0.85	0.827	0.849	0.143	0.088
3 Three-factor model	967.077	149	6.497	0.837	0.812	0.836	0.149	0.172
4 Three-factor model	1,045.944	149	7.02	0.822	0.794	0.821	0.156	0.143
5 One-factor model	2,190.161	152	14.409	0.595	0.542	0.593	0.233	0.192

*n = 249. Model 2 merges perceived overqualification and psychological capital, Model 3 merges perceived overqualification and person-organization fit, Model 4 merges perceived overqualification and knowledge hiding, and Model 5 merges all variables*.

### Descriptive statistics

Table 4 illustrates the correlation coefficients and other statistics of constructs, which can preliminarily forecast the correlation coefficient. There are positive relationships between POQ and PsyCap (*r* = 0.64, *p* < 0.01); PsyCap and KH (*r* = −0.52, *p* < 0.01); POQ and KH (*r* = −0.49, *p* < 0.01).

**Table 4:** Results of descriptive statistics analysis.

**Construct**	**1**	**2**	**3**	**4**	**5**	**6**	**7**	**8**	**9**	**10**	**11**
1: Gender	1										
2: Age	0.032	1									
3: Education	0.024	0.060	1								
4: Work years	0.012	0.845**	0.175**	1							
5: Industry type	−0.116	0.155*	−0.035	0.105	1						
6: Salary level	0.052	0.414**	0.422**	0.426**	0.093	1					
7: Marital status	−0.066	0.105	−0.121	0.141*	0.062	0.018	1				
8: POQ	−0.032	−0.096	0.008	−0.132*	−0.071	−0.068	0.011	1			
9: PsyCap	−0.003	−0.101	0.159*	−0.052	−0.088	−0.022	0.089	0.638**	1		
10: P-O fit	−0.054	−0.126*	0.042	−0.121	0.021	0.067	−0.073	−0.162*	−0.260**	1	
11: KH	−0.048	−0.080	0.060	−0.058	0.146*	0.117	−0.088	−0.490**	−0.516**	0.553**	1
*M*	1.320	2.440	2.140	2.430	2.360	2.610	1.550	2.962	2.920	3.058	2.903
SD	0.468	0.999	0.984	1.145	1.253	0.910	0.615	0.958	0.880	1.061	0.983

n = 249. *p < 0.05; **p < 0.01. POQ, perceived overqualification; PsyCap, psychological capital; P-O fit, person-organization fit; KH, knowledge hiding.

### Hypothesis test

We used Amos 26 to test the relationship between variables, and the results show where the fit index of each variable was acceptable (see Table 5). Additionally, structural model fit index was also acceptable: (χ^2^ = 298.497, *df* = 146; χ^2^/*df* = 2.044; IFI = 0.970, TLI =0.964, CFI =0.970, RMSEA = 0.065, RMR = 0.048).

**Table 5:** Overall summary of structural models.

	**Unstandardized estimate**	**S.E**.	***T*-value**	***P***	**Standardized estimate**	**Hypothesis**	**Judgement**
POQ → KH	−0.325	0.091	−3.574	***	−0.274	H1	Yes
POQ → PsyCap	0.688	0.073	9.451	***	0.659	H2	Yes
PsyCap → KH	−0.401	0.089	−4.522	***	−0.352	H3	Yes

POQ, perceived overqualification; KH, knowledge hiding; PsyCap, psychological capital; P-O fit, person-organization fit. ***p < 0.001.

As predicted, POQ has a significant positive association with PsyCap (β = 0.688, *p* < 0.001), which indicated the direct effect of POQ on PsyCap was confirmed. Therefore, H2 is supported. In addition, PsyCap was related negatively to KH (β = −0.401, *p* < 0.001), and POQ also had a negative association with KH (β = −0.325, *p* < 0.001), which indicated preliminarily the existence of indirect effect (see Table 5). Therefore, H1 and H3 are also supported.

We used Hayes's (2017) Macro Process model for mediation analysis. Hayes' PROCESS (5,000 bootstrapping was specified) was used to test hypothesis 4 (see Table 6 for the results). The mediation hypothesis in this paper was confirmed by the prominent indirect effect (indirect effect = −0.224, 95% CI = [−0.321, −0.128]). Thus, psychological capital mediated the correlation between POQ and KH, thus, Hypothesis 4 was also accepted.

**Table 6:** Overall summary of mediation effects.

**Mediation path**	**Effect**	**Boot SE**	**95% CI**		**Decision**
			**Boot LL**	**Boot UL**	
POQ –> PsyCap –> KH	−0.224	0.05	−0.321	−0.128	Partial mediation

**Figure 2:**
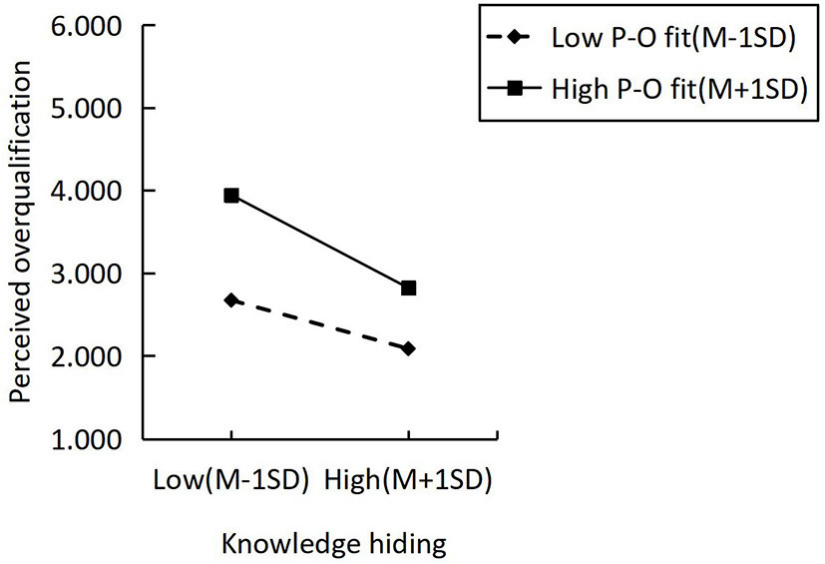
Moderating effects and simple slope test.

Following Hayes and Andrew (2009), we apply PROCESS 3.4 to examine the moderating role of person-organization fit on the relation between POQ and KH by 5,000 bootstrap samples. We also ran Process Model 1, in which the P-O fit moderated the association between the POQ and KH. The consequences in Table 7 showed interaction item moderating negatively the relationship between POQ and KH (β= −0.552, LLCI = −0.216, ULCI = −0.049). To analyze further the moderating effects, in Figure 2, we used a simple slope test to observe the strength of the negative correlation between POQ and individual KH at high and low levels of P-O fit, respectively. As shown in the figure, the negative correlation between POQ and KH is slow when the P-O fit is low. When P-O fit is higher, it enhances the negative correlation between POQ and KH, indicating that when individuals' P-O fit is high, POQ will reduce KH behavior. Therefore, H5 is supported.

**Table 7:** Overall summary of moderation effects.

**Variables**	**B**	**SE**	**β**	***t***	***P* (2-tailed)**	**95% CI**		
						**LL**	**UL**	
POQ	−0.041	0.130	−0.040	−0.316	0.752	−0.298	0.216	
P-O fit	0.866	0.139	0.934	6.226	0.000	0.592	1.139	
POQ × P-O fit	−0.133	0.042	−0.552	−3.135	0.002	−0.216	−0.049	
*R* ^2^								0.491
Δ*R*^2^								0.020

*POQ(X); P-O fit(W); KH(Y). POQ, perceived overqualification; KH, knowledge hiding; P-O fit, person-organization fit*.

## Discussion

In the rapidly developing information age, the significance of knowledge is becoming prominent. As an indispensable component of knowledge studies, knowledge hiding has crucial implications for both academia and practitioners. The importance an organization attaches to knowledge management and intangible assets largely determines the success and sustainable development of the organization; at the same time, for individuals, organization members regard knowledge as a significant asset (Zhu et al., 2019), and their expertise and unique knowledge can bring social and economic value, prompt them to improve their position, and gain political authority (Ma and Zhang, 2021). Therefore, we aim to explore the interaction between the potential positive aspects of employee perceived overqualification and employee knowledge hiding and to discuss the effect of psychological capital and person-organization fit on employee knowledge management behavior. For the relationship between the two key variables discussed in this paper, some researchers have analyzed the direct and indirect effects between them (Ma and Zhang, 2021; Khan et al., 2022), while others have proposed a U-shaped relationship between the perceived overqualification and individual work behavior (Woo, 2020). Although people are more and more interested in knowledge hiding in the context of the organizational crisis caused by COVID-19, the relationship between the two key variables in this paper has not been fully studied so far. This study starts with the process of examining the positive role of the perceived overqualification and combines the existing views to explore the benefits of individuals with a high level of the perceived overqualification on the knowledge management of organizational individuals. We consider this relationship to be linear and propose hypotheses about the relationship between the perceived excess of qualification and knowledge hiding based on the self-classification theory and the self-regulation theory. This study samples Chinese enterprises and produces the following results: First, there is a simple linear relationship between individuals' perceived overqualification and knowledge hiding. Perceived overqualification has a potential positive impact, which is highly conducive to reducing individuals' knowledge hiding behavior. Second, psychological capital plays a partial mediation role in the negative correlation between the perceived overqualification and knowledge hiding. Third, individual organizational fit can promote the positive effect of the perceived overqualification on individual knowledge management behavior, that is, individual organizational fit can enhance the negative association between the perceived overqualification and knowledge hiding.

The results of this study highlight the importance of the perceived overqualification as an antecedent of knowledge concealment behavior in knowledge management. Understanding the mental state of “additional abilities” that individuals have can assist us to inform and benefit managers on how to better promote perceived qualified individuals to decrease knowledge hiding. For another, the consequences of this research differ in part from some previous results in the existing literature. The present findings suggest a linear relationship of a negative correlation between the perceived overqualification and knowledge hiding. Based on self-classification theory and according to self-regulation theory, the perceived overqualification leads to beneficial results (Ma et al., 2019; Zhang J. et al., 2020). In comparing these different conclusions, we need to clarify several key points. First, we chose the potential positive aspects of the perceived overqualification to study its relationship with knowledge hiding, which more accurately measures the individual's psychological ability, rather than treating the perceived overqualification as a unified negative state; second, the samples and timing varied between studies. This study evaluates the attitudes of 249 Chinese business individuals toward perceived excess qualification and knowledge hiding in the context of COVID-19. Finally, the study has a different focus. Given the results presented in previous studies, this study mainly investigates the effect of positive effect of the perceived overqualification on knowledge concealment, that is, perceived overqualification can effectively reduce employees' knowledge hiding behavior, and there is a linear negative correlation between the two, while the main aim of Ma and Zhang (2021) and Khan et al. (2022) is to validate the linear positive correlation between the perceived overqualification and knowledge hiding.

Furthermore, this study also found that psychological capital as an intermediary can effectively influence knowledge hiding behavior, psychological capital often manifested as a positive mental state, where individuals are optimistic, hopeful, resilient, and self-effective, able to effectively respond to various workplaces and give a positive attitude to the future of work (Walters, 2010; Paek et al., 2015). This psychological state often makes individuals reduce their negative attitudes, which is conducive to the perception of employees with excess qualifications to shape their positive psychological cognition, thereby reducing the probability of knowledge hiding behavior. Instead, they work in the form of mutual help and initiative. This study extends the potential active impacts of the perceived overqualification and examines how psychological capital enhances the positive effects of the perceived overqualification on reducing knowledge hiding behaviors in terms of positive mental states, thus responding to the call for potential positive effects on the perceived overqualification and filling a key gap in the literature.

Finally, we also analyzed the impact of person-organization fit on the relationship between the perceived overqualification and knowledge hiding. Existing literature points out that person-organization fit is also a key element affecting individual knowledge management behavior, and person-organization fit is the similarity and compatibility among individuals and organizations (Kristof-Brown et al., 2005). An important aspect associated with it is the values and atmosphere of the organization. When employees perceive excess qualifications, if the values of employees and the organization are highly consistent, the regulatory role of people-organization fit will be reflected in the organizational atmosphere of incentive and support, and promote employees to actively reduce knowledge hiding behavior. The results show that, according to our expectation, the association between the two main variables in this paper is modulated by the person-organization fit, making the passive effect of the perceived overqualification on knowledge hiding enhanced when the person-organization fit is higher. Although the seniority level of individual employees incorporates knowledge management is mixed and not all individuals can be described in a positive mental state, the atmosphere, culture, and values of the organization will provide us with information and teach us how to better reduce their knowledge hiding behavior.

### Theoretical contributions

First, this research increases the literature on hidden knowledge from the subjective perspective of employees and expands the scope of knowledge hiding in the knowledge management system. The researchers acknowledge that knowledge hiding is an important factor requiring high attention and that some attention has been given to its impact based on employee perceived overqualification. These studies provide important insights into the perceived excess qualification and knowledge hiding, namely that the perceived overqualification is one of the main reasons behind this factor. The existing literature lacks new empirical evidence for the existence of a negative intrinsic link between the perceived overqualification and knowledge hiding. Our study also helps to extend the knowledge about the perceived overqualification through its findings. Based on our results, employees identified their KSAs as an essential asset they bring to the workplace and the organization, and their surplus qualifications are valuable additions to the organization. Because of their initiative in the team or organization, they may bring greater benefits to the team or organization. This study shows new empirical evidence for the existence of a causal relationship between the perceived overqualification and knowledge hiding. Our work responds to the academic call for more research on the potentially positive aspects of the perceived overqualification and the reasons for knowledge hiding and the consequences of these practices (Hu and Liden, 2015; Luksyte and Spitzmueller, 2016; Lin et al., 2017; Deng et al., 2018; Van Dijk et al., 2020).

Second, this study has made an important contribution to knowledge hiding by testing the mediation of psychological capital and the moderation of person-organization fit. Previous studies have rarely considered their predecessor, and in the past have examined knowledge concealment with working conditions, executive behavior, or situational factors. The subjective experience of employees' perceived overqualification has done less research on knowledge hiding, and overqualification is a common concern of organizations today. It is crucial to focus on this factor, as employee perceived overqualification is closely related to work status. The research on knowledge hiding is especially lacking in terms of potential positive aspects of the perceived overqualification. Our study found that when employees perceived overqualification, due to strong role self-efficacy and positive psychological cognition, employees will respond to teams or organizations by seeking more active or broader ways, that is, psychological capital will effectively affect employees' knowledge hiding behavior to a certain extent. From the theoretical perspective of person-organization fit, knowledge hiding is reduced when employee values align with organizational values. Due to psychological capital, this study enriched the research on the influencing factors of employee subjective perspective “POQ” on knowledge hiding, confirming that the perceived overqualification is negatively linked with knowledge hiding.

Third, we also examined a moderated factor, “P-O fit” when addressing the negative effect of the perceived overqualification. Person-organization fit can relieve the positive impact of the perceived overqualification on knowledge hiding. By studying the moderated function of person-organization fit, we provide a novel and important condition for the effect of the perceived overqualification on employee psychology and performance (Bari et al., 2020). Our findings show that employees working in a team or organization with a better atmosphere and meeting their values can decrease the passive impact of the perceived overqualification, and also have a positive impact on their psychological capacity building.

### Managerial implications

Our research sheds new light on the consequences of overqualification for business practitioners. First, our findings suggest that overqualification is not the relative deprivation of overqualification and waste of talent. Employees can use their excess KSAs to temper their psyches and do more. They achieve self-efficacy by accomplishing a wider range of tasks, and they can actively make pro-social changes (Grant and Ashford, 2008; Parker and Collins, 2010). Overqualification will not only stimulate their sense of value in the team, organization, and society, and drive them to better job performance and respect for others. We stand for that the outcome of the perceived overqualification may vary; it depends on one's mind and attitude. That is, whether an individual wants to be an outstanding person of the general population or an ordinary person among the best group. When an employee perceives overqualification, he/she may conduct more job-seeking behaviors (Maynard and Parfyonova, 2013). In this study, we think that employees who perceive overqualification will pay more attention to their residual ability rather than insufficient working conditions, and are more likely to take the opportunity to take the initiative to complete more tasks, especially in an atmosphere full of organizational support, employees with greater psychological capital ability and higher levels of person-organization fit have greater probabilities to perform better.

First of all, from the perspective of the potential positive role of the perceived overqualification, this study discusses the reasons for the inconsistency between employees' perceived overqualification and employees' active work behavior in the existing research, which has a certain practical guiding value. It is suggested that managers should pay attention to employee selection and post allocation, fully understand employees' career development goals, analyze their post adaptability, and evaluate whether the enterprise can meet employees' needs and expectations.

Second, this study proposes the mediation role of psychological capital in the path between employees' sense of excess qualification and employees' knowledge hiding behavior. Based on social embeddedness theory, it verifies the influence process mechanism of “perceived overqualification—psychological capital—employees' knowledge hiding behavior”, which enriches the research results of the relationship between psychological capital and knowledge hiding behavior. Given that psychological capital plays an important role between employees' perceived excess qualification and knowledge hiding, it is suggested that enterprises with the problem of employees' excess qualification should take how to build employees' psychological capital and positive psychological cognition as the management focus. For example, arrange work carriers for employees with excess qualifications that can demonstrate their qualifications, release their qualification energy, and meet the expected benefits of qualifications to stimulate their knowledge sharing behavior. According to employees' unique abilities and psychology, through professional training and development plans, guide employees to continue to move in the right direction, and make full use of their qualifications to achieve the best interests of the team or organization.

Third, our results show that person-organization fit positively regulates the relationship between excess qualification and knowledge hiding at the individual level. Accordingly, enterprises should attach importance to employees' recognition of enterprise values and corporate culture, as well as the construction and maintenance of enterprise organizational atmosphere, pay full attention to, support and trust employees' contributions, create a psychological safety atmosphere for employees, provide them with opportunities to display their talents, and encourage and support employees' extra job behaviors beneficial to the organization to enrich their current work. So as to strengthen the identification of employees with excess qualifications with the organization, and then make employees show more positive behaviors.

### Limitations and future research directions

This paper has several limitations that should be resolved in future studies. First, since all the main variables in the study were fully self-reported in the form of a questionnaire since knowledge concealment is a more sensitive and negative topic, respondents may not give completely true responses due to social desirability bias. Therefore, we expect future non-self-reported, more credible ways to investigate perceived excess of qualification and knowledge concealment. Second, the types of the perceived overqualification vary with each individual. According to Maynard (2011), common studies all assume that employees want to use their KSAs to obtain a higher salary. However, some people may receive far less for their interest or sense of mission than they can be paid for their potential abilities. Maltarich et al. (2011) have proposed the concept of “intentional mismatch”, which proves that many people make different choices about their work because of their different needs. The perceived overqualification does become a deprivation in most cases, but in certain situations the potential positive effects of the perceived overqualification can produce excellent results. Third, we found that employees' psychological capital plays an intermediary role in the covering relationships and this can be further examined and validated on different samples across various cultural backgrounds in future research. Meanwhile, we investigated the moderating effect of person-organization fit on the relationship between the two key variables, and confirmed its active effect. In addition to person-organization fit, future researches can explore the other situations where the beneficial effects of the perceived overqualification are ignored or highlighted. In addition to the situational variables and employee personal psychological factors variables in this study, future studies can examine more personal characteristic variables or bias variables as moderators or mediators from the perspective of managers to gain insight into how managers capture potential positive outcomes of the perceived overqualification.

## Conclusion

This paper expands the literature on perceived overqualification, knowledge hiding, psychological capital, and person-organization fit, and verifies the inactive impact of the perceived overqualification on knowledge hiding. Our research contrasts with the predominantly negative narrative and empirical evidence on employees' perceived overqualification. Results show that perceived overqualification reduces employees' knowledge hiding, which is further regulated by person-organization fit, and increases the negative correlation between perceived overqualification and knowledge hiding. Apart from this, psychological capital also enhances this negative impact. As empirical studies of how potential positive aspects of the perceived overqualification affect knowledge hiding are still lacking, there are many development opportunities in this field. This study will serve as the basis for a future study of how the perceived overqualification of positive information affects employees' work behaviors in a variety of work settings.

## Data availability statement

The raw data supporting the conclusions of this article will be made available by the authors, without undue reservation.

## Author contributions

SW and WT organized the collection and preparation of data. JZ, YZ, and FL performed the statistical analyses, wrote the first draft of the manuscript, and made changes to the manuscript during the interactive review stage. ZZ provided us with substantial raw data access and cases for analysis. All authors contributed to the conception, design of the study, read, edited the manuscript, and suggested improvements at several stages during the preparation and revision of the manuscript.

## Funding

This study was supported by the National Natural Science Foundation of China (Award Nos. 71762033 and 71663058) and the National Social Science Foundation of China (Award No. 17BGJ036).

## Conflict of interest

The authors declare that the research was conducted in the absence of any commercial or financial relationships that could be construed as a potential conflict of interest.

## Publisher's note

All claims expressed in this article are solely those of the authors and do not necessarily represent those of their affiliated organizations, or those of the publisher, the editors and the reviewers. Any product that may be evaluated in this article, or claim that may be made by its manufacturer, is not guaranteed or endorsed by the publisher.
